# Evaluation of clinical outcomes of one-stage anterior and posterior surgical treatment for atlantoaxial tuberculosis complicated with neurological damage

**DOI:** 10.1186/s12891-019-2539-7

**Published:** 2019-04-06

**Authors:** Biao Wang, Rongan Shang, Tong Yang, Haiping Zhang, Huimin Hu, Wei Hu, Dingjun Hao

**Affiliations:** 1 Department of Spine Surgery, Honghui Hospital, Xi’an Jiaotong University College of Medicine, No. 76 Nanguo Road, Xi’an, 710054 Shaanxi China; 2 Department of Orthopaedics, Baoji Hospital of traditional Chinese Medicine, No. 43 Baofu Road, Baoji, 721000 Shaanxi China

**Keywords:** Spinal tuberculosis, Atlantoaxial, Neurological impairment, Surgical approach

## Abstract

**Background:**

Surgical treatment is mainly used for atlantoaxial tuberculosis with neurological damage. However, the anatomic structure around the atlantoaxial joint is complex, and the position of vertebral body is deep, which increases the difficulty of the operation and it is challenging for the surgeon to develop surgical strategy. The purpose of this study was to evaluate the clinical outcomes of one-stage combined anterior and posterior surgical treatment approach for atlantoaxial tuberculosis with neurological impairment.

**Methods:**

From January 2005 to January 2015, 12 patients suffering from atlantoaxial tuberculosis with neurological impairment were surgically treated by one-stage combined anterior and posterior approach. Preoperative CT scanning and MRI imaging showed unilateral or bilateral lateral mass destruction of the atlas, and varying destruction degrees of odontoid process, loss of atlantoaxial stability, and tuberculosis focus into the spinal canal resulting in the corresponding spinal cord compression in all patients. The preoperative neurological classifications were Class C for 4 cases, D for 8 cases according to the American Spinal Injury Association (ASIA) system. Quadruple sensitive anti-TB drug treatment was used in all 12 patients preoperative and postoperative. Patients’ clinical symptoms and neurological function recovery were evaluated by comparing the Visual Analogue Scale (VAS) score, Neck Disability Index (NDI), Japanese Orthopedic Association (JOA) score and ASIA grading before operation and at the final follow-up.

**Results:**

Mean surgical duration was 263.3 ± 43.6 min. Intraoperative blood loss was averagely 529.2 ± 169.8 ml. The average fusion period was 7.3 ± 1.5 months. No instrumentation loosening, migration or breakage was observed during the follow-up of 6.5 ± 2.9 years. The VAS, NDI and JOA scores were significantly changed to 1.00 ± 0.95, 9.50 ± 3.34 and 15.42 ± 1.44 at last follow-up (*P* < 0.05). The neurological function of all 12 patients was recovered to Class E according to the ASIA grading system.

**Conclusion:**

In the treatment of atlantoaxial tuberculosis with neurological impairment, one-stage combined anterior and posterior surgical approach have the ability to complete debridement and decompression, and reconstruction of the stability of the upper cervical spine.

## Background

Upper cervical tuberculosis refers to tuberculosis in C1–2 segment of spinal tuberculosis, therefore it is also known as atlantoaxial tuberculosis. This disease is relatively rare in clinic, and its incidence is only about 0.3–1% in total tuberculosis of spine [1–5]. In recent years, the incidence of tuberculosis is increasing worldwide, developing countries and with resurgence tuberculosis in North America and other developed countries in immunocompromised patients, the incidence of spinal tuberculosis, including involvement of the atlantoaxial, also is increasing [6–9]. Anatomically, this site is the transitional region of craniovertebral junction, at which the stress is more concentrated. Lesions can lead to the loss of the stability of upper cervical spine, atlantoaxial dislocation, giant paravertebral abscess, or even neurological damage and other symptoms, which are harmful to patients. The atlantoaxial anatomy is special, and the lesion site of tuberculosis is deep. Patients’ early symptoms are not typical. Most patients present with only neck pain and discomfort. About 30–70% patients often have symptoms of neurological dysfunction before treatment, since they did not receive timely treatment or had missed diagnosis or misdiagnosis [10, 11]. At present, surgical treatment is mainly used for atlantoaxial tuberculosis complicated with neurological damage. However, the anatomic structure around the atlantoaxial joint is complex, and the position of vertebral body is deep, which increases the difficulty of the operation and it is challenging for the surgeon to develop surgical strategy. In order to solve these challenges, multiple surgical methods have been developed. To the best of our knowledge, the surgical treatment of atlantoaxial tuberculosis combined with neurological impairment has rarely been reported. This article retrospectively reviewed the 12 cases of atlantoaxial tuberculosis complicated with neurological damage who received one-stage anterior and posterior surgery in our hospital from January 2005 to January 2015, to discuss the clinical prognosis of this operation.

## Methods

### General information

From January 2005 to January 2015, 12 patients with atlantoaxial tuberculosis complicated with neurological damage received one-stage anterior and posterior surgery. 7 of them were males and the other 5 were females, aging from 19 to 46 years old with an average of 33.3 ± 7.6 years old. All of these patients had persistent neck pain, stiffness, limited mobility and other local symptoms. 7 patients were complicated with afternoon fever, night sweat, weight loss and other systemic symptoms of tuberculosis. Their average erythrocyte sedimentation rate (ESR) was 59.6 ± 31.1 mm/h, and the average C-reactive protein level was 51.7 ± 42.9 mg/l. Preoperative cervical CT and MRI showed that there was lateral bone destruction at unilateral or bilateral Atlas for all patients, different degrees of bone destruction at odontoid process, loss of atlantoaxial stability, and intraspinal tuberculosis, leading to compression of corresponding segmental spinal cord. MRI confirmed that all the 12 patients had paravertebral abscess and retropharyngeal abscess formation. ASIA grading of preoperative neurological function assessment was: 4 cases of grade C and 8 cases of grade D (Table 1).

**Table 1:** Preoperative demographic and clinical characteristics of the patients

Patients	ESR (mm/h)	CRP (mg/l)	ASIA grade
1	28	8.6	D
2	65	35.8	C
3	110	128.6	D
4	15	5.4	C
5	47	68.2	D
6	82	76.8	D
7	70	56.2	C
8	36	16.5	D
9	58	18.2	C
10	86	110.2	D
11	20	6.8	D
12	98	88.6	D

*ESR* erythrocyte sedimentation rate, *CRP* C-reactive protein

### Preoperative preparation

All patients received anti-tuberculosis treatment with continuous usage of isoniazid, rifampicin, pyrazinamid, and streptomycin for more than 2 weeks. They were all treated with continuous skull traction after admission, in order to 1) stabilize the spine and avoid secondary damage of spinal nerves; 2) make the intraoperative reduction of atlantoaxial dislocation convenient; 3) favor the focal exposure of anterior surgery. Meanwhile, the nutritional support of patients was used for the correction of patients’ anemia and hypoproteinemia. All patients underwent routine chest X-ray and chest CT examination to exclude active pulmonary tuberculosis.

### Surgical procedure

All 12 patients were treated with one-stage anterior and posterior debridement, bone graft fusion and internal fixation.

Anterior operation: all patients were at supine position with general anesthesia by tracheal intubation. The left or right incision at the neck was chosen according to side with more pus on the cervical MRI. The plane of angulus mandibulae was centered and used as the starting point for incision to make an arc incision of about 8 cm long along the medial border of the sternocleidomastoid muscle. The platysmamyoides were cut open, the cleidomastoids were pulled outward, and the anterior vertebral fascia was dissected by oblique upward blunt separation through the spaces between the carotid sheath and the tracheoesophageal sheath. The anterior vertebral fascia and the walls of abscess were cut open to suck the pus. The atlantoaxial joint was exposed to the side of head. Caseous necrosis, sequestrum and other inflammatory tissues were completely removed. Partial tuberculosis tissues were saved for pathological examination, tubercle bacillus culture and drug sensitivity test. Intraspinal tuberculosis lesions were carefully removed to completely relieve the spinal cord compression. After a thorough rinse with a small flush gun, streptomycin powder was sprayed locally, and streptomycin gelatin sponge was used to cover the front of the vertebra. The drainage tubes were indwelt, and the cuts were closed layer by layer.

Posterior operation: patients were turned over. Longitudinal incision was made in the middle of the posterior of neck occiput. The skin and subcutaneous tissues were cut sequentially. Subperiosteal stripping was made along the two sides of spinous process, to fully expose the cranial base, C1 posterior arch and C2–4 lamina. The tuberculosis lesions of 3 cases spread into the posterior arch of Atlas, affecting the rear structure and leading to its damage. These patients were treated with resection of posterior arch of Atlas, focal debridement, occipitocervical fixation and bone graft fusion with autogenous iliac bone. For the other 9 cases without tuberculosis spreading to the posterior structure of Atlas, only occipitocervical fixation and bone graft fusion with autogenous iliac bone were applied. For patients who received posterior focal debridement, streptomycin gelatin sponge was also used for local coverage after thorough rinse in order to control the local lesions.

### Postoperative treatment

Postoperative pathological examination confirmed that all 12 patients were infected with tuberculosis. They received routine antibiotics treatment for 48 h after operation, followed by 3 days of hormone and dehydration drug treatment. All patients were discharged after a stable condition. They were asked to wear a head and neck brace for 3 months after being discharged. For the 5 cases with negative tubercle bacillus culture results, quadruple strengthening antituberculosis therapy with isoniazid, rifampicin, pyrazinamide and streptomycin was applied for 3 months, after which streptomycin was replaced with ethambutol for a continuous treatment for 9–15 months. For the 7 cases with positive tubercle bacillus culture results, 4 sensitive antituberculosis drugs were selected according to the results of drug sensitivity for 12–18 months of treatment. For those who received streptomycin, it was also replaced by other sensitive drugs after 3 months for another 9–15 months of treatment. ESR, CRP, liver and renal function were reviewed monthly.

### Evaluation of curative effect

All patients were reexamined 3, 6, 9, and 12 months after operation, followed by reexamination every 1 year. The location of internal fixation, whether there was loosening or displacement, the status of bone graft fusion and so on were evaluated by X-ray imaging or CT scanning. The criteria of cure of tuberculosis were as follows: with more than 2 years follow-up, patient’s symptoms disappeared, no internal fixation failure and sinus formation happened, imaging confirmed that bone fusion was good, and 6 months of continuous ESR and CRP examinations were normal. Patients’ clinical symptoms and neurological function recovery were evaluated by comparing the Visual Analogue Scale (VAS) score, Neck Disability Index (NDI), Japanese Orthopedic Association (JOA) score and American Spinal Injury Association (ASIA) grading before operation and at the final follow-up.

### Statistical analysis

*t*-test was used to compare the VAS score, NDI score and JOA score before operation and at the last follow-up. Statistical analysis was performed by SPSS 19.0 (Chicago, IL, USA) software. *P* < 0.05 was considered to be statistically different. All statistical data were recorded as mean ± standard deviation.

## Results

**Fig. 1:**
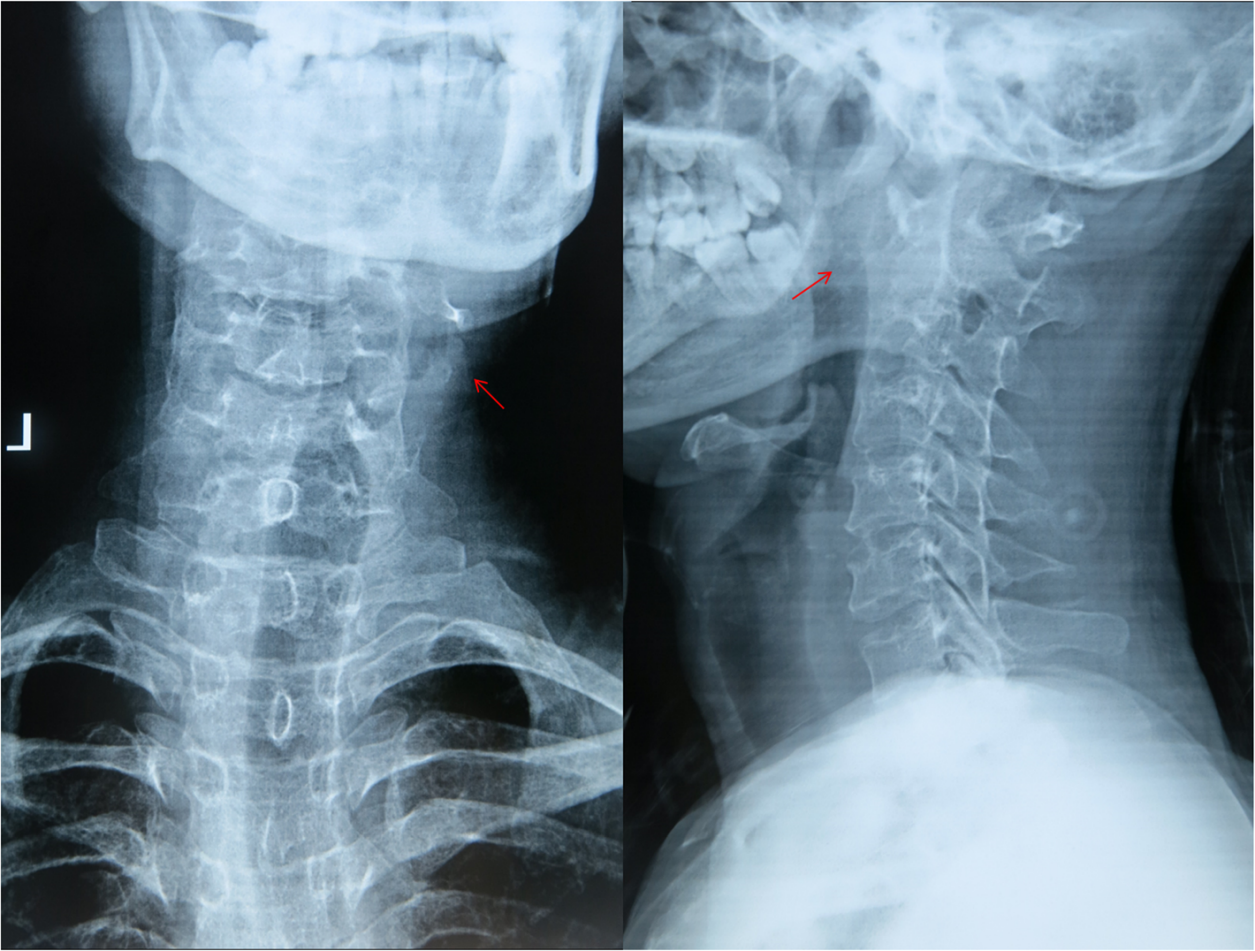
A male patient at 29 years old with atlantoaxial tuberculosis combined with neurological damage. The ASIA grading was C. Preoperative anteroposterior and lateral radiographs showed mild lateral curvature of the cervical spine and bone disorders of the upper cervical spine

**Fig. 2:**
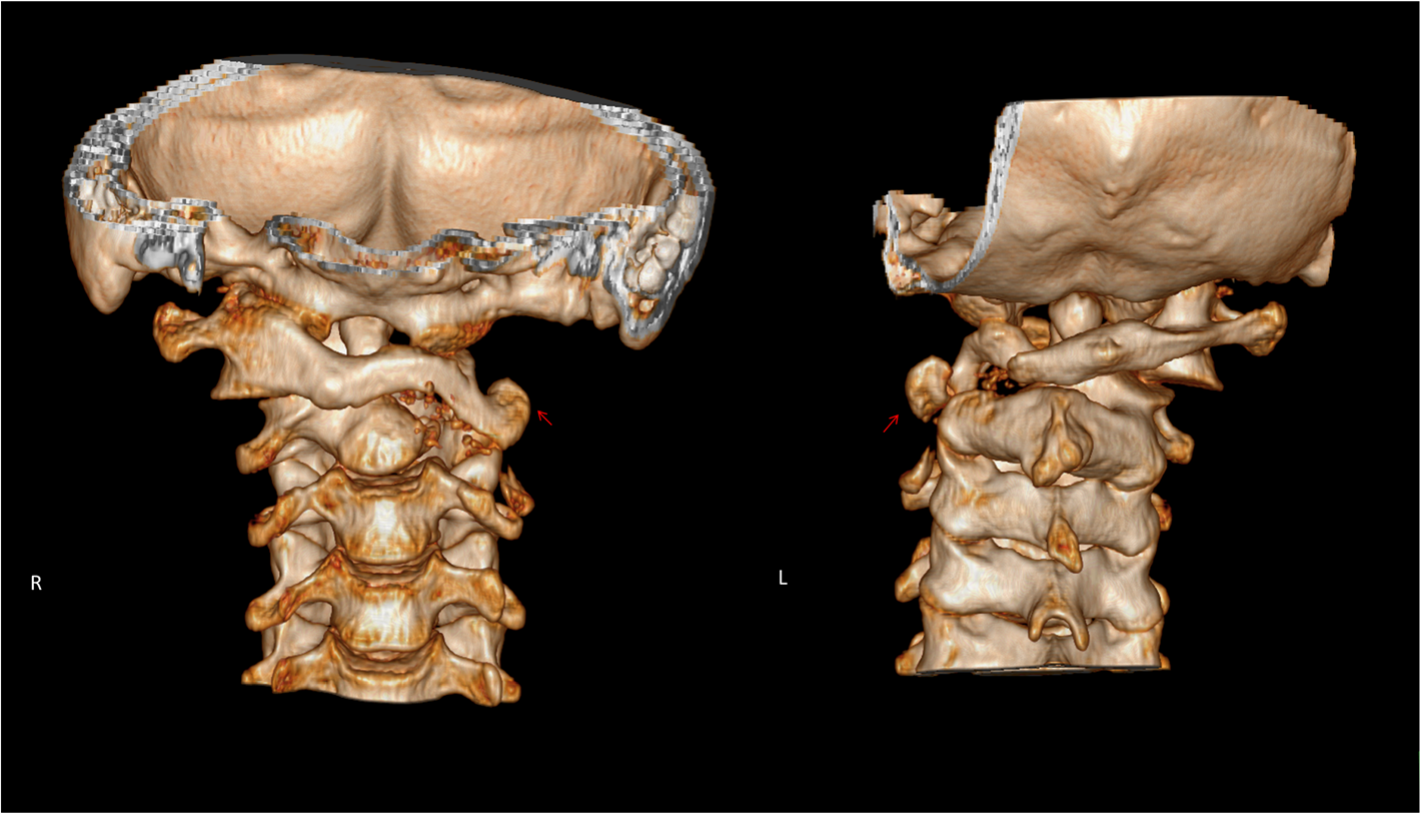
Preoperative 3D-CT examination revealed left lateral mass destruction of Atlas caused by tuberculosis lesion and the loss of atlantoaxial stability

**Fig. 3:**
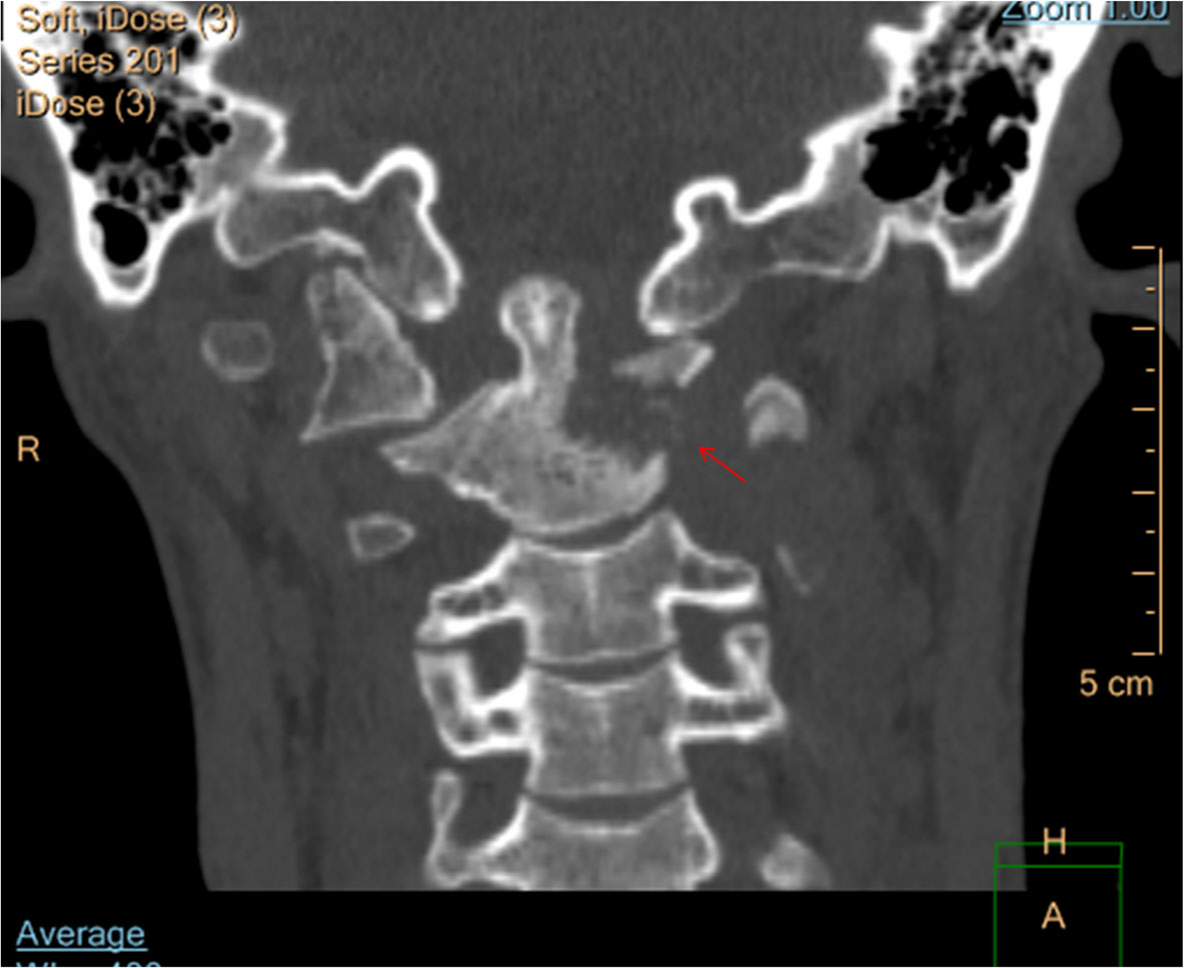
Preoperative CT scanning showed severe damages at the odontoid process and the vertebral body on the left side

**Fig. 4:**
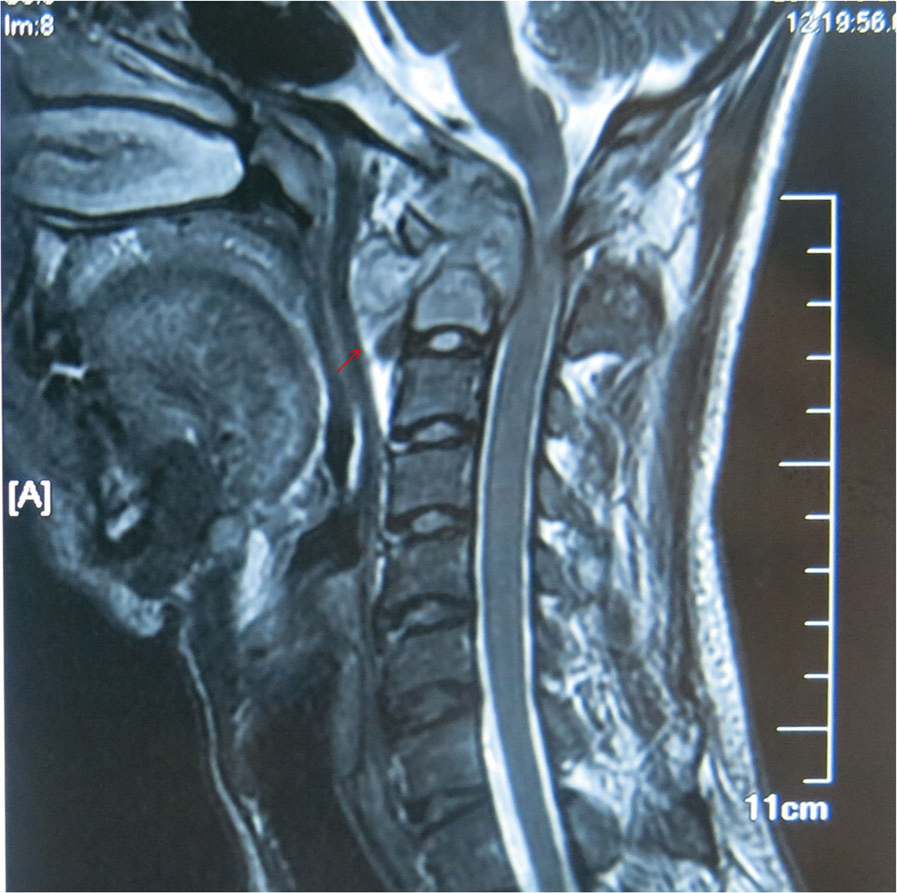
Preoperative MRI examination showed formation of atlantoaxial paravertebral abscess and retropharyngeal absess. The tuberculosis lesions spread into the spinal canal, leading to obvious compression of the spinal cord at C1–2 segments

**Fig. 5:**
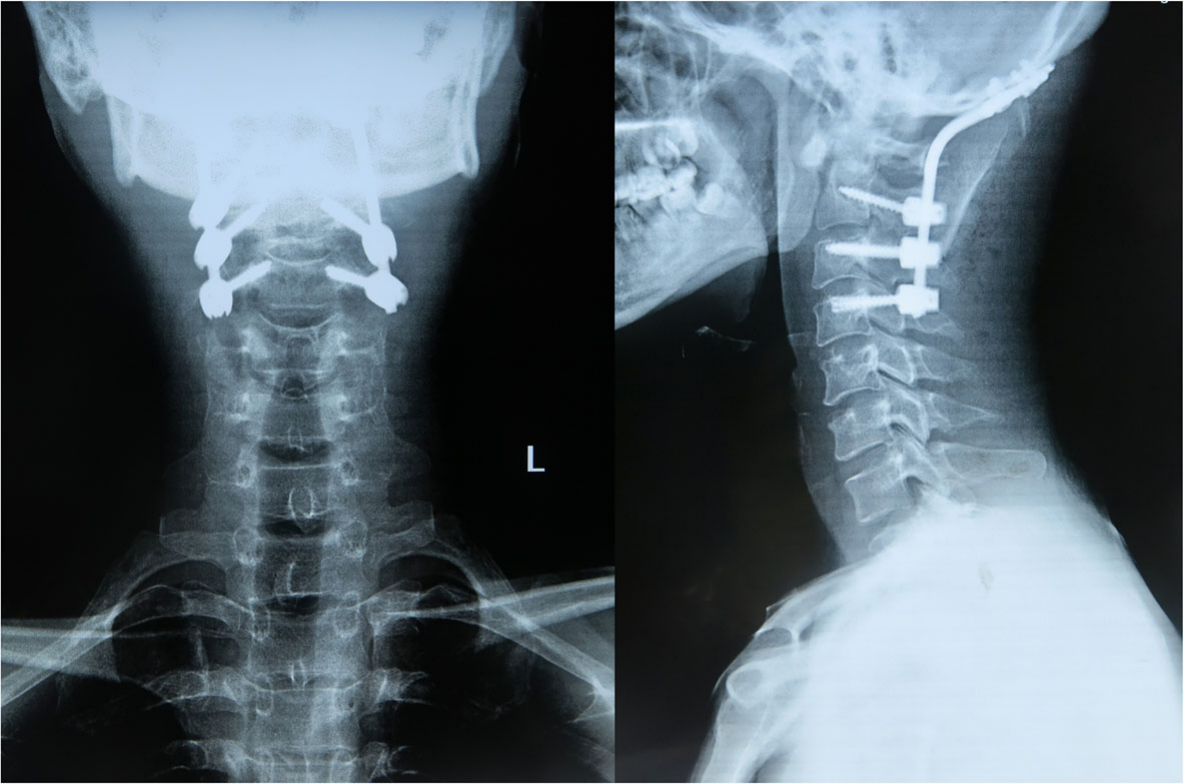
X-ray examination one week after operation showed good recovery of cervical physiological curvature, good internal fixation position, and satisfactory stability reconstruction of the upper cervical spine

**Fig. 6:**
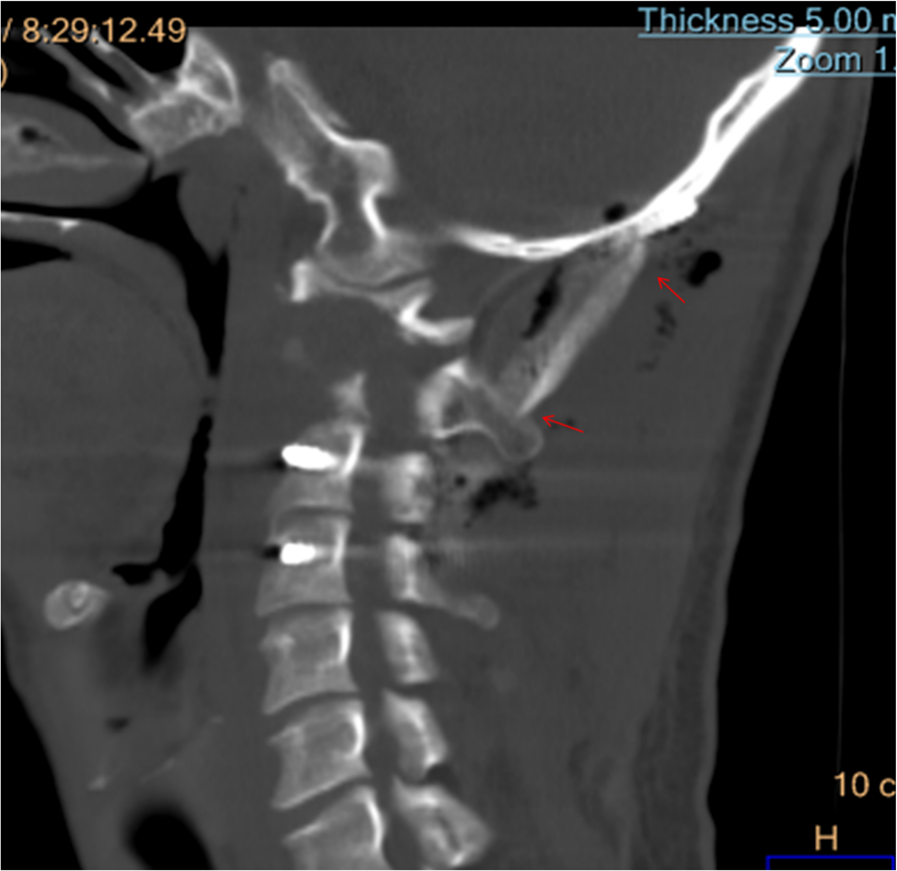
CT examination 9 months after operation showed occipitocervical fusion by posterior bone graft

**Fig. 7:**
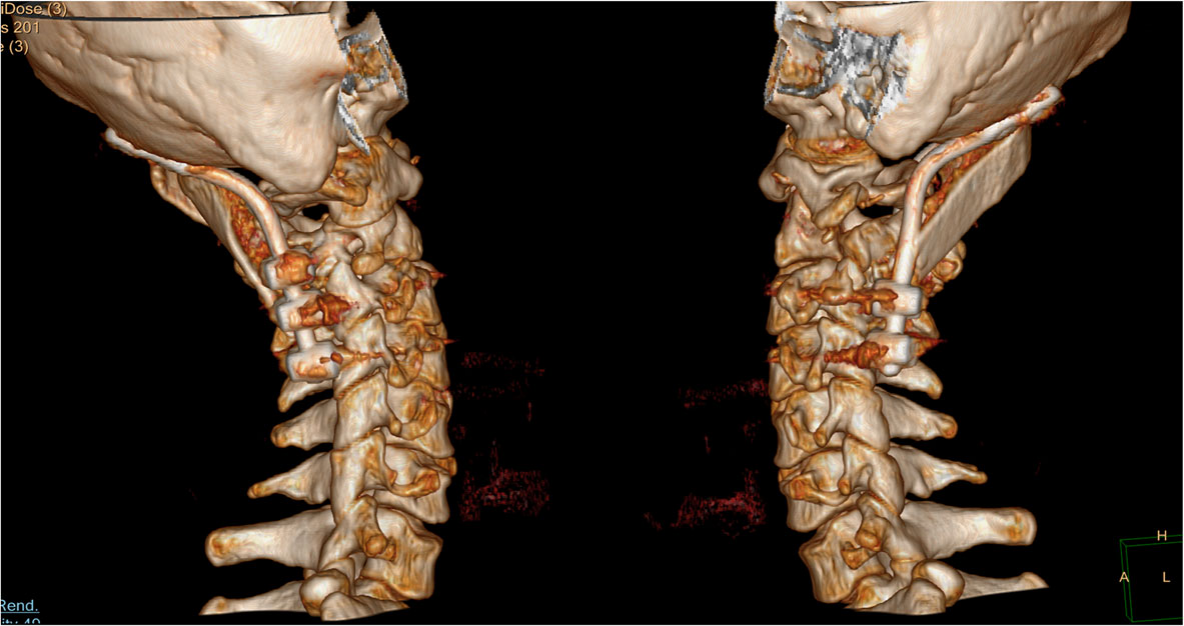
Lateral 3D-CT examination 5 years after operation showed that the internal fixation position was good, and the physiological curvature of the cervical vertebra was maintained well

**Fig. 8:**
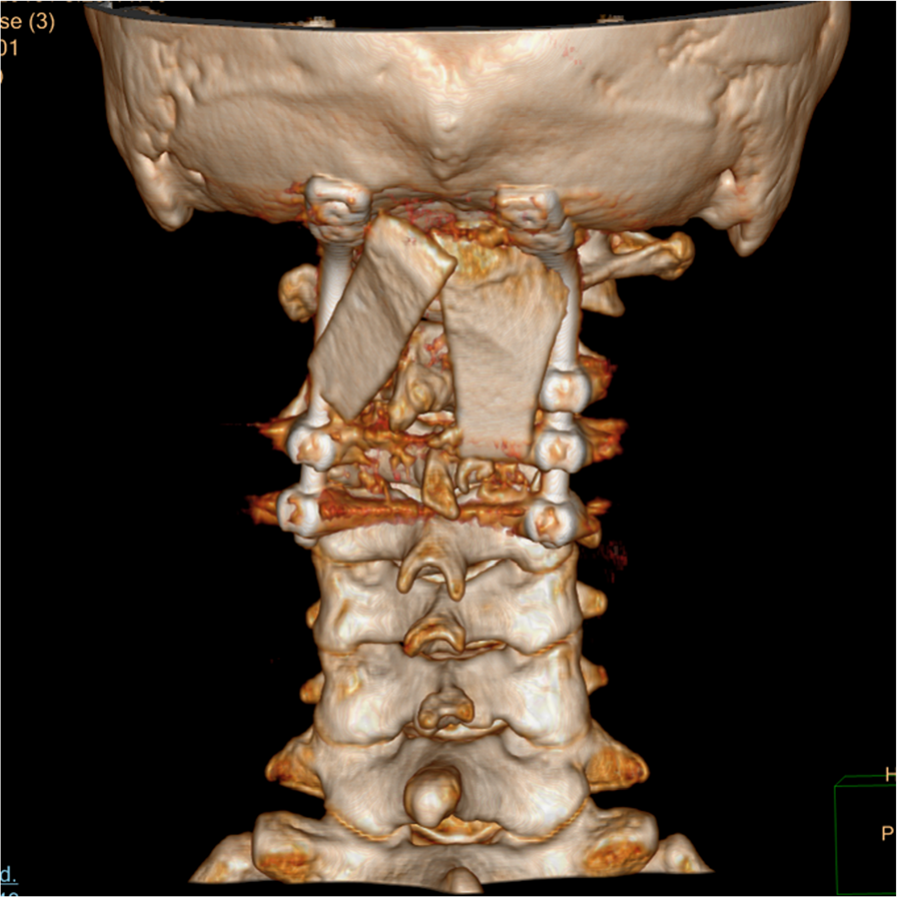
Posterior 3D-CT examination 5 years after operation showed that the internal fixation and bone graft position was good

The average operation time was 263.3 ± 43.6 min. And average amount of bleeding was 529.2 ± 169.8 ml. There was no serious complication such as vertebral artery or spinal cord injury. No cerebrospinal fluid leakage or esophageal leakage occurred. 1 patient had postoperative choking cough, suggesting great possibility of incomplete injury of superior laryngeal nerve. This was likely because of intraoperative traction of superior laryngeal nerve. This patient received symptomatic treatment such as neurotrophic therapy, etc. The symptom disappeared 3 months after operation. All other patients had no obvious surgical complications. The 12 cases were followed up for 2–12 years, with an average of 6.5 ± 2.9 years. CT results of 7 patients during the 6 months of follow-ups after operation showed that their occipitocervical fusions were complete. For the other 5 cases, they had complete occipitocervical fusions 9 months after operation shown by CT examinations. The average fusion period was 7.3 ± 1.5 months. No internal fixation loosening, displacement, fracture or other internal fixation related complications occurred. All the 12 patients were clinically cured at the final follow-up. The patients’ average VAS score at the final follow-up was 1.00 ± 0.95, which was significantly different from that before operation which was 7.08 ± 1.16 (*P* < 0.05). And the average NDI score at the final follow-up (9.50 ± 3.34) were also significantly different from that before operation (37.08 ± 2.64) (*P* < 0.05). In addition, the average JOA score at the final follow-up was 15.42 ± 1.44, significantly different from that before operation (8.08 ± 2.11) (*P* < 0.05). Detailed clinical results and statistical results are shown in Tables 2 and 3. The 12 patients had different degrees of neurological function recovery at the final follow-up, of whom the 4 at grade C and 8 at grade D were all improved to be grade E (typical case is shown in Figs. 1, 2, 3, 4, 5, 6, 7, 8).

**Table 2:** Clinical outcomes of the 12 patients

Patients	Operation time(min.)	Bleeding amount(ml)	Fusion period(mos.)	VAS score		NDI score		JOA score	
				Pre-	Post-	Pre-	Post-	Pre-	Post-
1	220	450	6	7	0	35	7	9	17
2	260	300	6	6	1	39	8	6	14
3	320	550	9	8	0	34	6	10	16
4	210	750	6	6	2	37	16	5	12
5	250	650	9	8	0	42	7	10	17
6	310	550	9	9	1	36	9	9	15
7	230	350	6	5	1	41	11	6	16
8	280	400	9	8	0	33	7	8	15
9	330	800	6	6	1	38	9	5	16
10	210	300	9	7	3	37	16	11	17
11	300	650	6	8	2	36	10	10	15
12	240	600	6	7	1	37	8	8	15

*Pre* Preoperative, *Post*- Postoperative

*VAS score* Visual Analogue Scale score, *NDI score* Neck Disability Index score

*JOA score* Japanese Orthopaedic Association score

**Table 3:** Statistical results in the VAS score, NDI score and JOA score

	Preoperative	Postoperative	95% Confidence interval		*P*-value
			Lower	upper	
VAS score	7.08 ± 1.16	1.00 ± 0.95	5.02	7.15	< 0.001
NDI score	37.08 ± 2.64	9.50 ± 3.34	25.09	30.07	< 0.001
JOA score	8.08 ± 2.11	15.42 ± 1.44	−8.43	−6.24	< 0.001

*VAS score* Visual Analogue Scale score, *NDI score* Neck Disability Index score

*JOA score* Japanese Orthopaedic Association score

Data are presented as mean ± standard deviation

## Discussion

Although tuberculosis is still an important health killer in developing countries, people from developed countries could not escape from it yet. Since the resurgence of tuberculosis in the United States in 1985, tuberculosis is spreading all over the world in recent years. The number of patients increases year by year [12, 13]. Nussbaum et al. [14] reported 75% of patients with spinal tuberculosis were diagnosed after 1983, which suggested the frequency of spinal tuberculosis is on increase. The incidence of spinal tuberculosis is relatively low, accounting for less than 1% in all tuberculosis, and accounting for only about 6% in extrapulmonary tuberculosis [6, 15]. Atlantoaxial tuberculosis is extremely rare in clinic, accounting for only about 0.3–1% of all spinal tuberculosis [1–5, 9]. Literatures about clinical study of this disease are relatively rare due to such a low incidence.

Atlantoaxial tuberculosis is often a secondary infection. The tuberculosis infection at the retropharyngeal space is a protracted course of disease, which spreads to the atlantoaxial joint, forming localized tuberculosis lesions. Of course, there are still very few patients with primary atlantoaxial tuberculosis, rather than secondary [16]. Occurrence of atlantoaxial tuberculosis is more concealing. The clinical symptoms appear relatively late, so that patients often have serious damage before treatment. Neurologic deficits are common seen in patients with tuberculosis of the atlantoaxial segments, which is associated with compression on the spinal cord or nerve roots [17]. Arunkumar et al. [11] reported that atlantoaxial tuberculosis complicated with neurological symptoms accounted for about 30–70%, and Gupta et al. [5] reported an even higher ratio of such disease at 80%. Atlantoaxial tuberculosis is more likely to combine with neurological damage as compared to spinal tuberculosis at other sites. Behari et al. [18] reported that the incidence of atlantoaxial tuberculosis complicated with neurological damage is higher than that of lower cervical tuberculosis. Hsu and Leong [19] reported thatatlantoaxial tuberculosis patients with neurological damage were 42.5%, significantly higher than the neurological damage rate of 15–30% at other sites of spinal tuberculosis.

Patients with atlantoaxial tuberculosis complicated with neurological damage often have formation of posterior pharyngeal wall and paravertebral abscess, serious damage of lateral mass of atlas, odontoid process or the vertebral body of axis, loss of atlantoaxialstability, intraspinal occupancy and compression of spinal cord. It is generally difficult to work with only conservative treatment. Surgical intervention to remove tuberculosis, full decompression, and reconstruction of upper cervical spine stability appear to be particularly necessary [20, 21]. However, it is still controversial for which surgical strategy to be used for the accomplishment of the above purposes at present. Single anterior or posterior operation is now rarely used. For single anterior operation, the tuberculosis lesion is often secondary to the front retropharyngeal space tuberculosis, the tuberculosis lesions were mainly located in the anterior part of the vertebral body, and the compression of spinal cord is usually caused by the from pressure. Therefore, anterior operation has the advantages of direct removal of lesions, direct decompression, and removal of spinal cord compression. However, due to the serious damage of atlantoaxial anterior column, the anatomical characteristics of atlantoaxial joint itself, and lack of reliable anterior internal fixation material making it difficult to get firm anterior fixation, and so on, it is particularly difficult to achieve the fusion of bone graft and the reconstruction of stability after focal debridement. Fang et al. [10] reported that single anterior operation has a more than 50% of the failure rate of bone graft fusion. Meanwhile, among the 12 cases in this study, the tuberculosis lesions of 3 cases spread into the posterior structure of the atlantoaxial joint, making it impossible for complete focal debridement by single anterior operation. Although single posterior operation can well reconstruct the stability of the upper cervical spine, the tubercolosis lesions and spinal cord compression mainly locate in the anterior column of the vertebral body, making it impossible to achieve focal clearance or decompression of neural canal. Zhang et al. [22] reported that treatment by single posterior focal clearance and short segment fixation and fusion for 11 cases of children with atlantoaxial tuberculosis had satisfactory effect, however, for patients complicated with neurological damage and patients with imaging of spinal cord compression, anterior decompression was often necessary. Therefore, the combined therapy with anterior and posterior operations has become the first choice for treatment of atlantoaxial tuberculosis complicated with neurological damage. On the basis of focal debridement and spinal cord decompression by anterior operation, posterior operation could not only further remove the lesions spread into the posterior column but also provide strong reduction strength for the dislocation or subluxation of the atlantoaxial joints and achieve reconstruction of the upper cervical spinal stability.

When the combined anterior and posterior operations are used, the main difference of posterior approach is the different fixation method. The surgeon can choose from atlantoaxial fixed fusion and occipitocervical fixed fusion. Under the premise of complete focal clearance of tuberculosis, short segment fixation and fusion should be used as far as possible for maximum reservation of activity of the cervical spine. To minimize the loss of cervical spine mobility is an important principle to choose the fixed fusion method [23]. Thereafter, atlantoaxial arthrodesis has more advantages. However, the disease progressions were late for the 12 cases in this study, which all developed with neurological symptoms and formation of prevertebral giant abscess causing serious damage of atlantoaxial joints. Preoperative CT examination showed that all the 12 cases were complicated with lateral mass destruction at unilateral or bilateral Atlas, making it impossible to imbed the Atlas pedicle screw or lateral mass screw. While the strength of the screw at posterior arch of Atlas is poor, unable to reconstruct the stability of the upper cervical spine, and the posterior arches of 3 patients were removed making it impossible to imbed screws. Therefore, occipitocervical fixed fusion was used in this study. We believe it is more important to reconstruct the stability of the upper cervical spine, as compared to mobility of the upper cervical spine.

Anterior operation can choose from transoral approach, lateral upper cervical approach, anterior retropharyngeal approach and traditional anterior cervical approach [6, 11, 18, 24–27]. Transoral approach is the most direct way for the exposure of the atlantoaxial anterior. However, the operation space of this approach was narrow through mouth, and the operative field was deep. In addition, it is easy to get mixed infection, recurrence of tuberculosis, change of pronunciation, edema of the tongue, leakage of cerebrospinal and other complications [28]. Meanwhile, since all the 12 cases in this study were complicated with neurological symptoms, making the perioperative oral nursing difficult, the transoral approach is not very suitable. Lateral upper cervical approach is another commonly used method to expose the atlantoaxial joints. However, this approach usually exposes only one side of the atlantoxial joint. For patients with extensive tuberculosis foci or abscess lesions on both sides, exposure at both sides is required for complete focal debridement [27]. This not only increases the surgical trauma, but also extends the operation time, increasing the surgical risk. At the meantime, the external jugular vein needs to be cut off during this operation when necessary, and the carotid sheath often needs to be cut open. In addition, this operation is adjacent to the vertebral artery, further increasing the surgical risk. The retropharyngeal approach can reveal the lower clivus, the C1 anterior arch, the C2 odontoid process and the vertebral body, which could avoid potential pollution of *Mycobacterium tuberculosis* and mixed infection in mouth and throat. However, the anatomical structure of this approach is complex, which often requires excision of the submandibular gland, and exposure of the hypoglossal nerve, facial artery and other important structures. The requirement of the surgical technic is high and there might be many complications. The traditional anterior cervical approach was used for all the 12 cases in this study. This procedure is an extension of the upper cervical spine of the classical Smith-Robinson approach. Its operation is simple, and most surgeons are familiar with this approach. This approach is safer since only the superior laryngeal nerve and hypoglossal nerve need to be protected at the operational site during the exposure, where there are no other important structures. However, the exposure of the atlantoaxial joint by this surgical approach is not as complete as the above operation methods. Since the mandibular block often requires the usage of pulling hook to stretch toward the head for the exposure of the Atlas, there might be risk of incomplete clearance of the foci. But for the 12 patients in this study, they received operations by this approach with the usage of small flush gun, which completely cleared the disease foci and decompressed the spinal cord. By medium and long term follow-up observation, there was no recurrence of tuberculosis, and all the 12 patients had neurological function recovery.

## Conclusion

In summary, for the treatment of atlantoaxial tuberculosis complicated with neurological damage, one-stage combined anterior and posterior surgical operation can achieve debridement of tuberculosis lesions, complete decompression of the spinal cord and reconstruction of the upper cervical spine stability. Traditional anterior cervical approach for anterior operation, combined with occipitocervical fixed fusion for posterior operation is a reasonable and reliable treatment strategy. In that case, good clinical outcomes will be obtained through medium- and long-term follow-up observation.

## Abbreviations

ASIA: American Spinal Injury Association
C: Cervical
CRP: C-reactive Protein
CT: Computed Tomography
ESR: Erythrocyte Sedimentation Rate
JOA: Japanese Orthopedic Association
MRI: Magnetic Resonance Imaging
NDI: Neck Disability Index
TB: Tuberculosis
VAS: Visual Analogue Scale
